# Next-Generation Wastewater-Based Epidemiology: Green Automation for Detecting 69 Multiclass Pharmaceutical and Personal Care Products in Wastewater Using 96-Well Plate Solid-Phase Extraction by LC-MS/MS

**DOI:** 10.3390/molecules30183694

**Published:** 2025-09-11

**Authors:** Bhaskar Karubothula, Veera Venkataramana Kota, Dnyaneshwar Shinde, Raghu Tadala, Vishnu Cheerala, Samara Bin Salem, Wael Faroug Elamin, Grzegorz Brudecki

**Affiliations:** 1 RASID Laboratory of Abu Dhabi Quality & Conformity Council (ADQCC) & M42 Environmental Sciences, Abu Dhabi P.O. Box 853, United Arab Emirates; vkota@m42.ae (V.V.K.); rtadala@m42.ae (R.T.); vcheerala@m42.ae (V.C.); welamin@m42.ae (W.F.E.); gbrudecki@m42.ae (G.B.); 2 Abu Dhabi Quality and Conformity Council UAE, Abu Dhabi P.O. Box 853, United Arab Emirates; samara.salem@qcc.gov.ae

**Keywords:** Pharmaceuticals and Personal Care Products (PPCPs), wastewater, LC-MS/MS, automated extraction, MAX SPE cartridges, method validation, greenness assessment

## Abstract

Conventional methods for detecting pharmaceutical and personal care products (PPCPs) in environmental samples are complex, resource-intensive, and not sustainable. Therefore, this study aimed to evaluate an automated sample preparation approach using the Biomek i7 Workstation to analyze 69 PPCPs in wastewater, with the objective to improve monitoring of public health and environmental protection. The method underwent extensive development, including optimization of UPLC-MS/MS parameters, preparation of wastewater matrix blank sample and assessment of extraction efficiency using three types of SPE cartridges. Extraction efficiency trials revealed that the order of suitability for SPE cartridges is Mixed-Mode Anion Exchange (MAX) > Mixed-Mode Cation Exchange (MCX) > Hydrophilic–Lipophilic Balance (HLB). The method demonstrated specificity for all targeted PPCPs, with the max interfering peak for 1, 7 Dimethylxanthine reaching 14.79% of the response at the target limit of quantification (LOQ). The method met ±20% matrix effect tolerance for 63 PPCPs, while 6 PPCPs showed signal enhancement. The 8-point procedural calibration curve prepared using automated robotic extraction has demonstrated linearity across the tested range. A spiking study at low (LQC), medium (MQC), and high (HQC) quality control levels (*n* = 6), repeated on three separate occasions, showed % RSD values within 20% and % recovery between 80 and 120%. The method met validation requirements, showed reliability in Intra-Laboratory Comparison, Blind Testing (BT) and received high ratings for greenness (Green Analytical Procedure Index, Analytical GREEnness) and practicality (Blue Applicability Grade Index).

## 1. Introduction

The global consumption of Pharmaceuticals and Personnel Care Products (PPCPs) has increased significantly in recent years. This growth is partly attributed to the global COVID-19 pandemic and the overuse of certain groups of drugs (non-steroidal anti-inflammatory drugs (NSAIDs), antibiotics, and antidepressants) [1,2]. Approximately 3000 compounds are used as pharmaceuticals, with annual production exceeding several hundred tons [3]. PPCPs are generally categorized into two groups: pharmaceuticals which include prescription, over the counter, and veterinary drugs used to prevent or treat various diseases and personal care products, which encompass substances used in soaps, detergents, masks, bleaches, dyes, deodorants, shampoos, perfumes, hair creams, and skin and dental care products [4,5]. Further, pharmaceuticals include various classes of drugs such as antibiotics (e.g., tetracyclines, sulfonamides, macrolides, fluoroquinolones, cephalosporins, beta-lactams and penicillins), NSAIDs, antihypertensives, antihistamines, antifungals, antacids, antidiabetic, and others. In humans and animals, 30% to 90% of orally administered pharmaceuticals are excreted as active substances via urine and feces [6]. The increasing use of pharmaceuticals has led to a corresponding increase in the release of pharmaceuticals and their metabolites into wastewater, which contributes to their widespread presence in the environment.

Continuous usage and discharge through multiple pathways like from pharmaceutical industries, hospitals, improper disposal, and inadequate wastewater treatment have led to varying concentration ranges of these compounds in water bodies [7]. Extensive research in municipal wastewater treatment plants (WWTPs) across the world has reported variable and incomplete removal of chemicals during treatment [4]. This incomplete removal is primarily because WWTPs are not specifically designed to eliminate diversified PPCPs, thereby serving as pathways for these substances to enter the environment [4]. Studies have reported the presence of PPCPs in water which specifically include antibiotics, analgesics, antipyretics, antimicrobials, antacids, antihypertensives, antifungals, and disinfectants [5]. Yang et al. reported frequent detection of sulfamethoxazole, primidone, Caffeine, and DEET ranging from 10 to 100 ng/L in the final effluent of an advanced wastewater reclamation plant in Gwinnett County [8]. An analysis of wastewater in Spain and northern Italy revealed the presence of 4-aminoantipyrine; beta-blockers, like atenolol; diatrizoic acid; paracetamol, irbesartan, valsartan, ofloxacin, sulfamethoxazole Ibuprofen, and Caffeine, and aspirin levofloxacin and carbamazepine [9]. A study carried out in Rome, Italy, highlighted the detection of carbamazepine, diclofenac, Ibuprofen, and gemfibrozil [9]. In addition, elevated concentrations of paracetamol and paraxanthine and high concentrations of carbamazepine and tramadol metabolites were identified in UK wastewater following treatment at WWTPs [9]. Latvia’s main wastewater plant exhibited the highest concentrations of Caffeine, paracetamol, ciprofloxacin, and Ibuprofen [9]. A review of studies across Asia, Europe, and North America found significant regional variations in influent concentrations, attributed to differences in usage patterns [10].

The PPCPs play a crucial role in enhancing human and animal well-being and life expectancy [11]. Despite their benefits, concerns remain about the continuous discharge of these substances into the environment. Ultimately, they enter the water cycle, posing a serious threat to human health and aquatic ecosystems. Further, the occurrence of PPCPs is of concern because they remain active even at trace concentrations. Studies have suggested that long-term exposure could cause allergies, antibiotic resistance, and an effect on the endocrine system. Several studies have identified a growing resistance pattern to antibiotics due to the increased use of existing antibiotics, posing the biggest threat to humans in the form of antimicrobial resistance (AMR). According to the World Health Organization (WHO), overuse and misuse of antimicrobials are the main drivers in the development of drug-resistant pathogens. AMR is one of the most pressing global health threats of the 21st century. A recent study projects that between 2025 and 2050, AMR will directly cause 39.1 million deaths [12]. These figures are alarming; there is a need for proactive surveillance and monitoring of multiclass PPCPs in wastewater.

Wastewater-Based Epidemiology (WBE) is a great tool that can provide evidence for existing data gaps, helping to expand understanding of this threat and support targeted interventions. The concept of WBE emerged from studies examining PPCPs in surface and sewage water resulting from human excretion [13]. Wastewater influent data offers valuable insights into community usage of PPCPs [14,15,16]. Additionally, WBE has been instrumental in tracking pharmaceutical consumption trends, with studies documenting changes in drug usage during the COVID-19 pandemic [15,17,18]. Currently, some PPCPs are listed on U.S. EPA Contaminants Candidate List (CCL) 3 and CCL 4, indicating their presence in public water systems and potential need for regulation under the Safe Drinking Water Act (SDWA) [19,20]. The European Union has established comprehensive monitoring protocols to assess the concentrations of various substances in water matrices and WWTPs. A list of substances to be monitored throughout the European Union was published by commission implementing decision (EU) 2022/1307 of 22 July 2022 (directive 2008/105/EC) [21].

There are several reported methods for detection of PPCPs in wastewater [22,23,24,25,26,27]. In 2007, the United States Environmental Protection Agency (U.S. EPA) published reference method: Method 1694: “Pharmaceuticals and Personal Care Products in Water, Soil, Sediment, and Biosolids by HPLC/MS/MS” [28]. In this method, the complete lists of targeted PPCPs were divided into acid and base fractions for extraction using two protocols. Further, target analytes in this method were divided into four groups. Groups 1, 2, and 3 are extracted under acidic (pH 2) conditions. Groups 1 and 2 are run in the positive electrospray ionization (ESI+) mode and Group 3 is run in the negative electrospray ionization (ESI−) mode. Group 4 is extracted under basic (pH 10) conditions and is run in the ESI+ mode. Further, a very high sample volume of 1000 mL must be passed through HLB 20cc 1 gm cartridge and concentrated to 3 mL. Also, the method used reagent water for preparing a calibration curve. Using reagent water for the calibration curve may overlook matrix effects, affect analysis accuracy, and result in broader acceptance criteria for quality control samples as seen in Table 12 of the U.S. EPA 1694 method [28]. Further, analysis must be conducted by separate chromatographic methods for negatively and positively ionized molecules due to non-availability of a mass spectrometer with the capability of analyzing compounds efficiently in polarity switching mode in one injection. The extraction of high sample volume and evaporative concentration of extraction solvent is laborious, time consuming, error prone, and unsafe for humans and the environment.

The need for larger sample volumes increases the workload and creates potential for manual errors. High-throughput analytical methods are essential for real-time analysis and effective WBE implementation and monitoring. The accurate and timely detection of PPCPs in municipal wastewater presents considerable analytical challenges, particularly when dealing with multiclass compounds that have diverse physicochemical properties and fluctuating wastewater matrix compositions. Therefore, advancing WBE requires the development of sensitive, robust, and eco-friendly analytical techniques capable of handling high sample throughput, providing reliable data across a wide range of pharmaceutical substances, and ensuring data accuracy and consistency. In the present study, the system integrates automated solid-phase extraction (SPE) robotics with Ultra Performance Liquid Chromatography (UPLC) and a XEVO TQ-XS mass spectrometer, enabling the rapid and reliable analysis of up to 100 samples within 24 h. Sample extraction automation was achieved using the Biomek i7 modular sample preparation robotics, which performs tasks such as conditioning SPE 96-well plates, loading, cleanup, elution, and sealing plates. The UPLC system, coupled with a tandem quadrupole mass spectrometer, was employed to reduce chromatographic run time while maintaining high sensitivity and specificity.

The objective of this study was to develop a comprehensive, fully automated, high-throughput method for accurate analysis of PPCPs in wastewater, including 9 fluoroquinolones, 8 macrolides, 6 penicillins, 9 sulfonamides, 15 tetracyclines, 3 NSAIDs, 4 antihypertensives, 2 antifungals and 13 other PPCPs from different chemical classes. Additionally, the study aims to evaluate the method performance using key validation parameters such as linearity, limits of quantification (LOQ) and limit of detection (LOD), specificity, matrix effects, precision, accuracy, robustness, Intra-Laboratory Comparison (ILC), and Blind Testing (BT). Furthermore, the method was assessed for its eco-friendliness and practicality using tools like the Green Analytical Procedure Index (Complex MoGAPI), Analytical Greenness (AGREE), and Blue Applicability Grade Index (BAGI). After a thorough evaluation of extraction efficiency and validation, the analytical method was finalized to cover 69 analytes. To the best of the authors knowledge, this is the first comprehensive study to develop, evaluate, and validate a fully automated method for the analysis of 69 multiclass PPCPs, while also demonstrating its environmental sustainability and practical applicability through a greenness assessment.

## 2. Results and Discussion

### 2.1. Outlines of Method Optimization and Validation

The MRM method optimization was performed by infusing individual CRMs of targeted 69 PPCPs and 20 Internal Standards (ISTDs). Further, chromatographic method using UPLC was Optimized to finalize the column and mobile phase compositions. Thereafter, extraction efficiency was evaluated using three different 96-well plate SPE cartridges (HLB, MAX, and MCX) to compare targeted analytes elution profiles, and extraction efficiencies. Initial sample extraction protocol was optimized using manual negative pressure SPE extraction manifold in ASTM type I water and then in wastewater matrix. Manually optimized SPE protocol was transferred to Biomek i7 automated sample preparation workstation. The fitness for purpose of the automated extraction protocol was evaluated by validating it on LC-MS/MS against standard validation parameters. Measurement uncertainty (MU) was calculated at the targeted limit of quantification (LOQ) using international guidelines JCGM 100:2008 and EURACHEM/CITAC Guide CG 4 [29,30]. The validated method was further proved for its accuracy by participating in Intra-Laboratory Comparison (ILC) and Blind Testing (BT) as per ISO/IEC17025:2017, clause no: 7.7.1 j and k, respectively [31]. Method greenness and practicability was evaluated by using online software tools like AGREE, Complex MoGAPI, and BAGI [32,33,34].

### 2.2. Outcome of Method Optimization

For developing MRM method, parent and daughter ion scans were performed by infusing 100 ng/mL tuning solution of each analyte at a flow rate of 20 µL by combining tuning solution with mobile phase using combining mode functionality. All source-dependent and compound-dependent parameters like desolvation temperature, desolvation gas, cone gas, cone voltage (CV), and capillary voltage were carefully optimized for better sensitivity. After selecting the precursor [M + H]^+^ ion, collision energy (CE) was ramped to select specific product ions. Two of the macrolides, Azithromycin and Roxithromycin, have two charge states, ([M + H]^+^ and [M + H]^2+^) [35]. All these ions were crosschecked for their sensitivity and specificity. There were no noticeable differences in terms of sensitivity and selectivity; therefore, protonated mass as the precursor ion was selected. Further, it was observed that two isobaric compounds (Flumequine and Oxolinic Acid) from the fluoroquinolones class exhibited identical quantifiers; however, they are chromatographically separated by a minute. From the group of Tetracyclines, 10 compounds exhibited common ions (461.0 (4 Epianhydro Chlortetracycline, Anhydro Chlortetracycline, and Oxytetracycline), 479.3 (4 Epichlortetracycline, Anhydro Chlortetracycline, and Isochlortetracycline), 427.2 (4 Epianhydrotetracycline and Anhydrotetracycline), 445.5 (4 Epitetracycline and Tetracycline)) as shown in Table 1. However, all these Tetracyclines were well separated chromatographically. Efforts were made to improve method specificity/selectivity for all these isomeric compounds, by selecting distinct daughter ions and separation by chromatography for accurate quantification.

The U.S. EPA Method 1694 was originally developed to analyze 74 PPCPs. It was observed during the method development that for five compounds, namely Triclosan, Metformin, Sulfanilamide, Codeine, and Triclocarban, alternative analytical approaches are required to achieve satisfactory performance. This was further evident from the broad acceptance ranges specified in U.S. EPA Method 1694 for these compounds (quality control IPR—Initial Precision and Recovery, OPR—Ongoing Precision and Recovery). Triclosan exhibited greater sensitivity when analyzed using Atmospheric Pressure Gas Chromatography coupled with Tandem Mass Spectrometry (APGC-MS/MS) compared to LC-MS/MS, consistent with its GC-amenable nature reported in multiple studies [36,37]. Codeine, in contrast, showed good extraction recovery using the MCX cartridge [38]. Metformin, due to its highly hydrophilic nature, exhibited poor retention on the MAX cartridge, necessitating the use of ion-pairing reagents to enhance retention on both the extraction and separation columns, consistent with previous reports [29,30,39]. Sulfanilamide and Triclocarban demonstrated reproducibility issues when extracted using the MAX protocol, highlighting limitations in method robustness for these analytes. For these two compounds, the acceptable % recovery ranges in U.S. EPA Method 1694 are IPR: 6–170% and 55–108%, and OPR: 5–189% and 50–120%, respectively [28]. Due to these analytical challenges, the five compounds were ultimately excluded from the targeted list in this study.

During the initial method optimization studies, it was observed that several commonly prescribed and used PPCPs such as Norfloxacin, Acetaminophen, 1, 7 Dimethylxanthine, Caffeine, Cotinine and Carbamazepine were detected in the pooled wastewater sample used for preparing the matrix blank. Similar detections were reported in the recently published research papers [3,35,40,41,42,43]. A cartridge-passed pooled wastewater sample was analyzed using an LC-MS/MS system to check the efficiency of cartridge cleanup for pre-detected analytes. It was observed that this approach effectively removed pre-detected analytes from wastewater samples. This screened, blank wastewater matrix sample was utilized to prepare procedural calibration curves (CC) and quality control (QC) samples for method validation and for routine analysis. The primary objective of preparing a screened blank control matrix is to closely mimic real wastewater samples and account for matrix effects. In contrast, most reported studies and conventional methods use reagent-grade water to prepare calibration curves and QC samples, thereby overlooking the significant impact of matrix components leading to signal suppression or enhancement.

The trials conducted for the evaluation of extraction efficiency revealed that there is no clear order of suitability for different cartridges (MCX, HLB, and MAX) for the targeted 69 PPCPs. Comparable results were reported in many of the reported publications, as many articles reported that the method of analysis is primarily optimized for illicit drugs, though using MCX cartridge is also suitable for PPCPs [44]. It was observed from the data that out of 414 data points (for intensity in terms of average area and % RSD of 69 PPCPs extraction using three cartridges), results for area and % RSD were least preferred on 39 occasions for MAX, 43 occasions for MCX, and 58 occasions for HLB cartridges. Also, we tried to evaluate on how many instances results were first/second highest (average area) and lowest (% RSD) to check the most suitable cartridge for analyzing these 69 PPCPs in single extraction chemistry. It was observed that the most preferred results were observed for MAX (99 occasions), followed by MCX (95 occasions) and HLB (80 occasions). It was observed from the data in Table 1 that all three tested cartridges showed quantifiable area at the MQC level of concentration with acceptable precision (less than 20% RSD, *n* = 4) except for two compounds by HLB (Norgestimate (21.45) and Anhydro Chlortetracycline (23.79)) and MCX (Erythromycin (20.72) and Chlortetracycline (41.20)). It was observed from the data that all compounds were meeting acceptable precision (less than 20% RSD) using MAX cartridges, with the maximum % RSD of 16.02% for Virginiamycin. Consequently, the MAX SPE cartridge chemistry was selected for further development and validation of the method.

**Figure 1:**
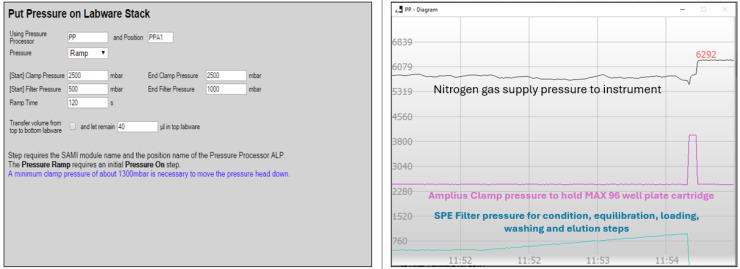
Pressure gradient profile for simultaneous extraction of 96 wastewater samples.

Optimized extraction protocol was subjected to further validation on a Beckman Coulter Biomek i7 automated workstation using a MAX 96-well plate cartridge for 69 PPCPs (Table 2). A sample volume of 4.5 mL was processed using the programmed extraction protocol steps as shown in Table 2 on the Biomek i7 system. Conditioning, equilibration, and washing reagents were added to the 96-well plate using the Thermo Fisher Multidrop Combi dispenser with a selection valve. Conditioning and equilibration steps were performed using 1.0 mL 100% MeOH and 1.0 mL of 100% ASTM type I water, respectively, to activate the active sites of stationary phase and to remove impurities for optimal interaction with the sample. Optimization of the pressure gradient proved crucial, as conditioning, equilibration, loading, washing, and elution steps had a significant impact on analyte elution and recovery. It was observed from initial trials that a pressure gradient starting from 500 millibar and ramping to 1000 millibar within 120 s using Amplius positive pressure extractor (Figure 1) was best suitable for recovering all targeted multiclass analytes. Target analytes were eluted into the collection tray using elution solvent and subsequently analyzed by LC-MS/MS. All peripheral instruments on the automated workstation were seamlessly integrated with the Biomek i7 and all functions were controlled using Biomek Automatic Workstation software (version 5.1).

### 2.3. Method Validation

The specificity or selectivity of the method was evaluated for each of the MRM transitions by analyzing six replicates of the control sample, prepared by pooling pre-screened control samples from 100 different locations, along with a process blank and solvent blank. During initial specificity trials, maximum interference of more than 10% were observed (Table 3) for three compounds, namely Caffeine (10.52%), Anhydro Chlortetracycline (13.74%), and 1, 7 Dimethylxanthine (14.79%); however, these interfering responses are less than the acceptable criteria of 30% of the LOQ concentration for all 69 PPCPs [45].

Matrix effect (% ME) was evaluated during the initial stages of the method validation to select a suitable calibration approach for accurate quantification. The matrix effect was evaluated by comparing the response of targeted PPCPs in ASTM type I water and in extracted control matrix at LOQ concentration. The results from Table 3 showed that the method meets acceptance criteria for a matrix effect of less than 20% for 63 of the targeted PPCPs. The maximum signal suppression of −12.72% was observed in the case of naproxen. It was observed that six PPCPs were failing to meet acceptance criteria of less than 20% for matrix effect [45]. Minocycline (53.68%) was observed to be affected the most by matrix effect, followed by Erythromycin (38.29%), Cotinine (30.53%), Ranitidine (22.29%), Azithromycin (22.12%), and Ciprofloxacin (21.62%). This observation further demonstrates that plotting calibration curves using reagent-grade water or solvent is insufficient when targeting multiple PPCPs in wastewater, given the unpredictable and variable nature of matrix components in wastewater from different sources. Therefore, to ensure accurate analysis of the targeted PPCPs, a procedural calibration technique was adopted for quantitation as mentioned in method validation guideline [45].

The sensitivity of the method was estimated by calculating the LOD (Table 3) by multiplying the standard deviation of the lowest spike level tested by 3.3. The estimated LODs were observed to range from 0.003 µg/L to 0.703 µg/L for targeted PPCPs. The results in Table 3 showed that for 58 targeted PPCPs, the estimated LODs were less than 0.2 µg/L. This result proves the sensitivity of the method without the need for evaporative concentration and using a smaller processing volume of less than 5 mL required for high-throughput analysis when compared to 1000 mL in conventional method [28]. It was observed during the study that estimated LOD was high (more than 0.5 µg/L) for two compounds (Cloxacillin and Penicillin V), which may be because of their unstable nature in aqueous solutions and degradation due to change in pH, temperature, and light.

Based on the method sensitivity, it was observed during the method development trials that procedural calibration curves with five different concentration ranges were required to cover all targeted PPCPs. From the targeted 69 PPCPs, for 21 analytes, linearity range was started from 0.05 to 2.00 µg/L (Table 3), and for 11 analytes it was from 0.10 to 4.00 µg/L. For 10 analytes, it ranged from 0.50 to 20.00 µg/L and for 24 analytes the range was from 1.00 to 40.00 µg/L. A higher linearity range of 2.50 to 100.00 µg/L was observed for only 3 analytes (Cloxacillin, Penicillin V and Digoxin).

During the validation study, the linearity of method was assessed using an 8-point calibration curve for all compounds, as detailed in Table 3. It was observed from the validation trials that the average coefficient of determination (r^2^) (*n* = 3, from day 1, 2, and 3 validation data) of procedural calibration was higher than 0.980 for 58 (out of 58, for 41 analytes the r^2^ was more than 0.990). For the remaining 11 PPCPs, the average coefficient of determination (r^2^) ranged from 0.953 to 0.978. The lowest average coefficient of determination (r^2^) for Miconazole was 0.9527, with an average deviation in back-calculated concentration under 20% across all calibration curve points during validation.

**Figure 2:**
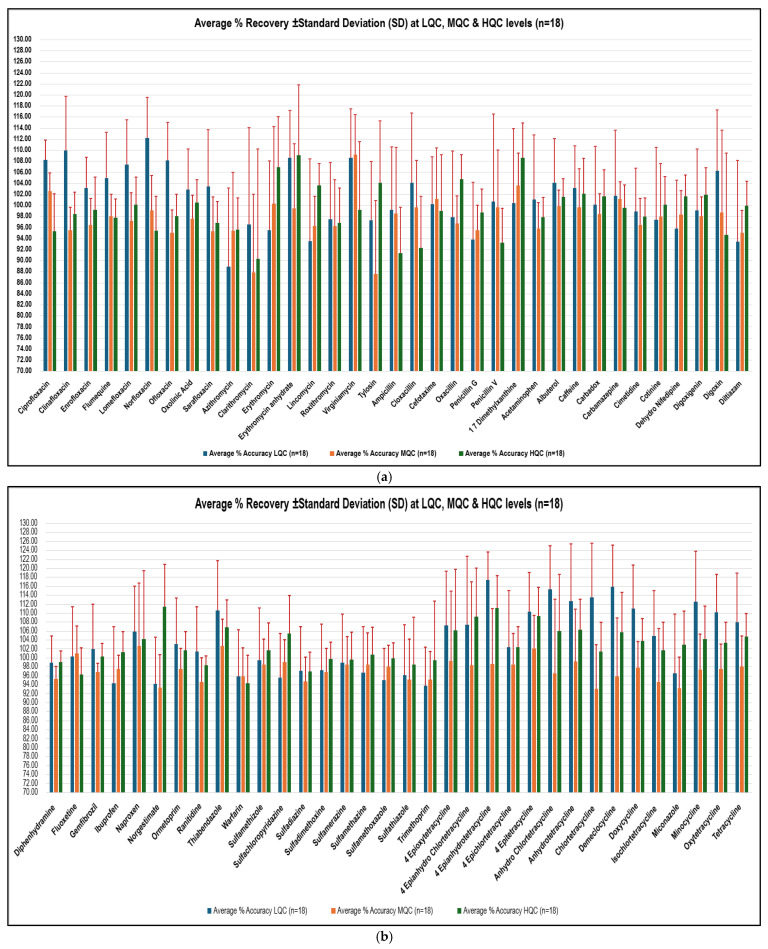
(**a**): Accuracy of the method in terms of Trueness (% recovery) and Precision (repeatability) for first 35 PPCPs; (**b**): accuracy of the method in terms of Trueness (% recovery) and Precision (repeatability) for remaining 34 PPCPs.

The estimated LOQ was determined theoretically by calculating 10 times the standard deviation of the lowest spiked level tested for each PPCPs (*n* = 6). It was observed that the estimated LOQs ranged from a minimum of 0.010 µg/L to a maximum of 2.131 µg/L (Table 3). In this work, the focus was to demonstrate that the lowest reported concentration meets requirements for the analysis at the detected limits in wastewater. Since no regulatory Maximum Limits (MLs) are defined for PPCPs in wastewater, the lowest sensitive and accurately quantifiable targeted LOQ concentration was assessed during method validation by evaluating the % RSD and mean % recovery. The data in Figure 2a,b show that at the targeted LOQs listed in Table 3, the method met the acceptable performance criteria for % recovery and % RSD across validation trials.

The accuracy of the method in terms of Trueness (% recovery) and Precision (repeatability) was evaluated by injecting six replicates of LQC, MQC, and HQC standards on three different occasions. The % RSD of 69 analytes were evaluated for compliance with acceptable method performance criteria of less than 20% [45]. Figure 2a,b shows error bars of % RSD on bar charts representing the data for average % recovery. It was observed from the data in Figure 2a,b that the method is precise and meeting the acceptable performance criterion at LQC, MQC, and HQC levels (*n* = 18) for all targeted PPCPs. The highest % RSD was observed for Clarithromycin (19.9%) at MQC and HQC levels. The average % recovery for all 69 targeted analytes were evaluated to ensure compliance with the acceptable method performance criteria of 70 to 130% [28]. The data presented in Figure 2a,b indicates that the average % recovery of the method consistently falls within the acceptable range for all PPCPs with exact values between 87.55% (Tylosin at MQC) and 117.31% (4 Epianhydrotetracycline at LQC) throughout the validations study.

The robustness of the method was assessed by examining its performance, under minor variations, as often occurs during routine analysis. The expected changes evaluated were analysis by different analysts and the use of different instruments. Two trials of the method validation were conducted on the instrument with AEQ ID 141 and one trial was performed on the instrument with AEQ ID 142 (internal identification code for Analytical Equipment (AEQ). Furthermore, two trials conducted on instruments with AEQ ID 141 were performed by two different analysts. It was evident from the results shown in Figure 2a,b that the method is robust, as it meets the acceptable performance criteria of mean % recovery and % RSD with most likely changes during routine analysis [28,45].

At all instances for 69 PPCPs, ion ratios were meeting acceptable performance criterion of 30%, with deviations ranging from a minimum of 1.11 for Warfarin to a maximum of 13.33 for Naproxen (Table 1). In the case of three PPCPs, namely Cloxacillin, Digoxin and Ibuprofen, sensitive and stable qualifier ion was not observed. Retention times (RT) for all analytes were observed to be consistent, with deviations of not more than ±0.2 min within the batch (Table 1). The Measurement Uncertainty (MU) of all analytes was calculated as shown in Table 3 and ranged from a minimum of 4.10 to a maximum of 22.96% at targeted LOQ for Warfarin (0.524 ± 0.021) and Virginiamycin (1.056 ± 0.242), respectively.

**Figure 3:**
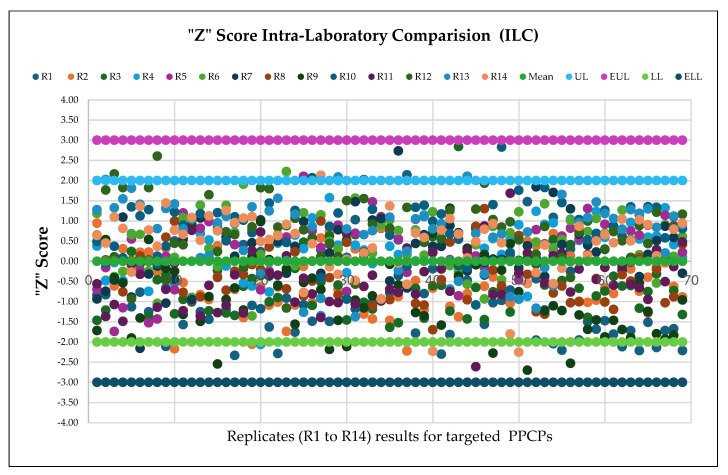
“Z” score Intra-Laboratory Comparison for targeted PPCPs.

**Figure 4:**
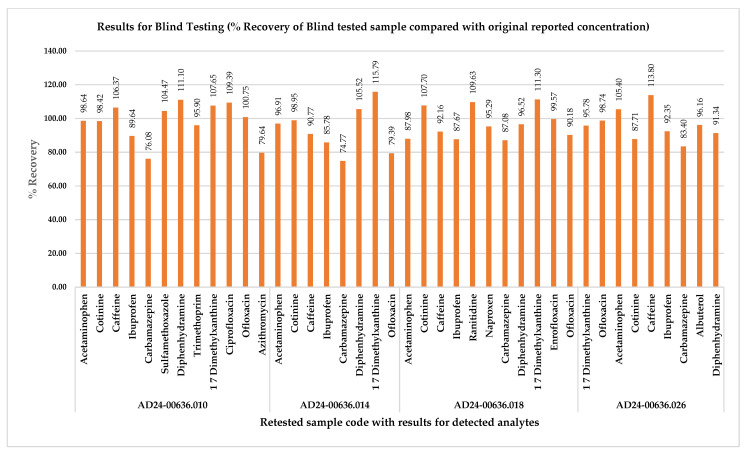
Results for Blind Testing (% recovery compared with original reported concentration).

 Intra-Laboratory Comparison (ILC) was conducted to assess the method performance across different settings (equipment and analysts), for ensuring results met acceptable criteria. Blank control samples, spiked at concentrations within the testing range (*n* = 7), were analyzed by two chemists. Intra-Laboratory Comparison results were evaluated by the lead scientist using “Z” score, calculated according to ISO 13528:2022, with Standard Deviation for Proficiency Assessment (SDPA) derived from calculated concentrations (*n* = 14, replicates from both analysts) [46]. Results were deemed acceptable with a “*Z*” score between −2 and 2, satisfactory between −3 and +3, and unacceptable outside this range. From the 966 data points (*n* = 14 for 69 PPCPs), 95.96% fell within the acceptable range (−2 to 2), and 4.03% were within the satisfactory range (−3 to 3), as shown in Figure 3. Blind Testing (BT) was conducted without analyst knowledge by rebooking and retesting the four previously reported positive samples. Sample identification was kept confidential and only accessible to the Quality Assurance manager until the BT evaluation was complete. Analysts used standard protocols, and the Quality Manager (QM) reviewed % recovery against original results. Results were considered acceptable with % recovery between 70% and 130%. The percentage recovery for analytes that tested positive in the BT round ranged from 74.77% for Carbamazepine to 115.79% for 1, 7 Dimethylxanthine in the sample coded AD24-00636.014, as illustrated in Figure 4. Further, BT results were evaluated for the Relative Percent Deviation (RPD) to compare two individual values meaningfully. The RPD ranged from a minimum of 0.217 (Enrofloxacin sample code AD24-00636.018) to a maximum of 14.43 (Carbamazepine in sample code AD24-00636.014).

### 2.4. Method Greenness and Its Practicality

**Figure 5:**
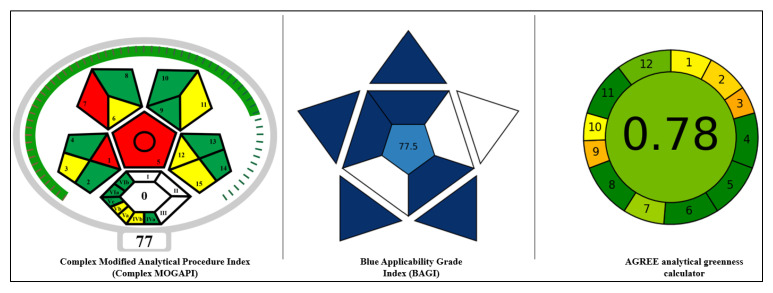
Evaluation of method greenness and applicability by MoGAPI, AGREE, and BAGI.

The tool, GAPI, visually represents the ecological impact and overall method greenness. In 2021, ComplexGAPI enhanced this framework by adding a hexagonal segment to assess activities beyond sample preparation and final analysis, considering parameters like recovery yields, processing conditions, green economy principles, occupational hazards, and E-factor for relevant methods. While it broadens sustainability evaluations, it lacks a unified scoring system for individual methods. To address this, a next-generation software, ComplexMoGAPI (v0.2) was used to simplify the assessment with a scoring system wherein the score of 100 represents the method is 100% green and excellent if greater than 75, acceptable if greater than 50, and not acceptable if less than 50. Parameters which are not applicable for the reported method like purification and E-factor were not considered in evaluations of ComplexMoGAPI [33]. As shown in Figure 5, combining pentagram and hexagram assessments, ComplexMoGAPI confirmed that the proposed method aligns with sustainability criteria, achieving an overall excellent eco-friendly score of 77 (https://mostwiedzy.pl/en/justyna-plotka-wasylka,647762-1/complexgapi (accessed on 19 December 2024)).

In AGREE, an analytical greenness calculator, results are displayed as a clock-like graph with the overall score and color representation shown at the center. This graph is based on 12 sections representing Green Analytical Chemistry (GAC) principles, each scored from one to zero. A score of one indicates a fully green practice, while a score of zero represents a non-green practice, indicated by red. The overall score is shown in the middle of the pictogram with values close to 1 and dark green color indicating that the assessed procedure is greener and eco-friendly [32]. As shown in Figure 5, for the reported method, eight principles were meeting the method greenness requirement; however, due to offline sample preparation and use of non-bio-based chemicals, the 1st and 10th principles were partially meeting the requirement of green analysis. However, the proposed method aligns with sustainability criteria and greenness score from AGREE, achieving an overall eco-friendly score of 0.78 (https://mostwiedzy.pl/en/wojciech-wojnowski,174235-1/AGREE? (accessed on 19th December 2024, git.pg.edu.pl/p174235/AGREE.)).

BAGI complements established green metrics and provides a quick overview of a method’s strengths and weaknesses regarding its applicability in laboratory settings. It evaluates ten core attributes, such as the type of analysis, the number of analytes assessed in a single method, the simultaneous analysis of multiple analytes, the analytical technique employed, sample analysis rate per hour, level of automation, reagents and materials used, instrument type, sample preparation process, and processing volume [34]. These inputs are used to create an asteroid-shaped pictogram for the proposed method with scoring from 25 to 100, where a high score of 77.5 indicates greater practicality of the reported method as shown in Figure 5 (https://mostwiedzy.pl/en/justyna-plotka-wasylka,647762-1/BAGI (accessed on 19th December 2024, git.pg.edu.pl/p174235/bagi)).

## 3. Materials and Methods

### 3.1. Materials and Reagents

The LC-MS grade formic acid was purchased from Sigma-Aldrich (Darmstadt, Germany) and 32% ammonia solution in water was purchased from Merck (Darmstadt, Germany). The MS grade methanol and acetonitrile were purchased from Honeywell (Charlotte, NC, USA). The ultrapure water (ASTM Type I) used was taken from Milli-Q IQ7015 (Millipore, Bedford, MA, USA). Solid-phase extraction (SPE) 6cc 500 mg cartridges (Oasis^®^ HLB, MAX, and MCX) used for blank control sample preparation were purchased from Waters (Milford, MA, USA). Oasis^®^ HLB, MAX, and MCX 96-well plates 60 µm (60 mg) were purchased from Waters (Milford, MA, USA) for checking extraction efficiency and selection of suitable chemistry to efficiently extract PPCPs from wastewater.

Reference standard solutions of all targeted 69 molecules with their 20 isotopically labeled Internal Standards (IS) were purchased from Dr. Ehrenstorfer (Augsburg, Germany) and Sigma-Aldrich ((Darmstadt, Germany) with purity of more than 99%. All the analytes were categorized into five groups based on instrument sensitivity and detection levels in wastewater. Standard stock solutions were prepared by using water, methanol, acetonitrile, and a combination of these three solvents based on their solubility and stability. Stock solutions of β-lactam antibiotics were prepared in a water–acetonitrile diluent, as these compounds are unstable in methanol and susceptible to methanolysis. [35]. Intermediate Mix (IM) standard solutions were prepared at 5 µg/L (group 1), 10 µg/L (group 2), 50 µg/L (group 3), 100 µg/L (group 4) and 250 µg/L (group 5) in methanol–acetonitrile (50:50 *v*/*v*) after applying the correction for the form (salt) and purity (%) of CRM. Further, these IM standard solutions were used to prepare the working calibration standards and quality control samples by spiking them in screened wastewater blank matrix (for blank matrix preparation). The mixture of deuterated Internal Standards working solution (10 to 40 µg/L) was prepared in methanol–acetonitrile (50:50 *v*/*v*) and spiked at fixed concentration before sample extraction. All standards and solutions were packed properly and stored in the dark at −20 °C.

### 3.2. Instrumentation

The Hamilton Microlab STAR M (Bonaduz, GR, Switzerland) robotic system was used for aliquoting test portions of 100 wastewater samples collected from various locations. The Hamilton robotic system was equipped with 4 channel pipette tool, auto decapper, and tube carriers for centrifuge tubes (50 and 15 mL). The aliquoting process was carried out using a pre-programmed method via the Venous 4 software (version 0.5).

Automated 96-well plate SPE was performed using the Biomek i7 Workstation (Indianapolis, IN, USA), equipped with 8-channel pipetting tool, alps for placing SPE 96-well plates, reservoirs, elution plates, and tube racks to load the sample tubes. The Modular Biomek i7 Workstation was equipped with positive pressure ALP Amplius extractor to perform SPE extraction, Thermo Fisher Multidrop Combi dispenser with guided selection valve to perform the SPE volume dispensing steps, and a4S 4titude automated role heat sealer to seal the elution plates. The complete instrument setup was controlled by the pre-programmed Biomek method launcher software (version 5.1). After the addition of ISTD and buffer, samples were vortexed using Benchmark^®^ Bench mixer multi-tube vortexer (Benchmark Scientific, Sayreville, NJ, USA).

Data were acquired using a Waters Acquity UPLC I class plus with a binary pumping system equipped with vacuum degasser, temperature controlled autosampler with Flow Through Needle (FTN) function, column oven, and coupled to a tandem quadrupole (QqQ) mass spectrometer (Xevo TQ-XS, Waters Corporation, Manchester, UK), with the Z spray ESI source operating both positive and negative polarity switching mode.

### 3.3. Mass Spectrometric and Liquid Chromatographic Method Optimization

Mass spectrometric method optimization was started by tuning individual PPCPs including their metabolites and Internal Standards on an XEVO TQ-XS mass spectrometer. The tuning solutions of individual CRMs were prepared at 100 µg/L. The tuning parameters including capillary voltage, cone voltage (CV), and collision energy (CE) were optimized for each analyte by infusing tuning solution in combined mode (tuning solution combined with mobile phase) for accurate selection of the tuning parameters. For initial trials, three to four product ions were selected due to matrix variability of wastewater samples then quantifier and qualifier ions were accurately selected for each analyte to get better selectivity and sensitivity. The segmented MRM method was created based on the retention times of analytes to get a minimum of 12 points per peak, for all MRM channels as shown in Table 1. The MRM method was created with polarity switching mode for both positive (+Ve) and negative ionization modes (−Ve) simultaneously. The optimized capillary voltage was 1.20 kV for positive mode of ionization and 2.80 kV for negative mode ionization. Nitrogen was used as nebulizer, desolvation, and cone gas, while argon was used as collision gas (Table 2).

The MRM method developed by direct infusion without chromatography, was further evaluated on ACQUITY Premier BEH C18 Column (130 Å, 1.7 µm, 2.1 mm × 100 mm) using Waters ACQUITY UPLC I Class Plus system with a XEVO TQ-XS mass spectrometer. The mobile phase composition was optimized through multiple trials with varying buffer strengths and organic modifiers to ensure effective ionization and sensitivity for both positively and negatively charged molecules. To optimize chromatographic conditions, a 100 ng/mL sample in wastewater and reagent water was used to check matrix interferences and separation. As demonstrated in Table 1, all 69 targeted analytes along with their Internal Standards (ISTDs) were eluted between 2.1 and 8.3 min. The remaining 3.7 min of run time were utilized for comprehensive column washing and equilibration before and after the elution of all analytes, to ensure the system was ready for subsequent injections.

### 3.4. Wastewater Matrix Blank Preparation Protocol

One of the most limiting factors in the analysis of routine wastewater samples was the absence of true blank wastewater matrix as commonly prescribed PPCPs are ubiquitously present in wastewater [35]. A review of the existing literature and standard methodologies highlights a predominant reliance on reagent-grade water for preparing linearity and quality control samples [11,22,47,48]. However, plotting calibration curves using reagent-grade water may compromise the accuracy of quantification, as it fails to account for matrix effects arising from the diverse compositions of wastewater collected from different locations. Due to matrix effects, routine wastewater sample analysis is often subject to significant interferences, typically in the form of ion suppression or enhancement. If a procedural calibration curve is not used to quantify PPCPs in wastewater, correcting for matrix effects and calculating absolute recovery are necessary. However, doing it daily for approximately 100 samples for targeted 69 analytes is not practicable, and can be inaccurate. To overcome this, matrix blank or representative wastewater sample was prepared by mixing real-time wastewater samples from distinct locations and passing it through a cartridge which can retain the analytes present in wastewater. This approach aims to prepare the closest possible matrix blank from the daily collected wastewater samples for calibration curve and quality control.

From the 100 wastewater samples collected across distinct locations, four 1-L samples were randomly selected and combined to prepare a 4-L blank matrix for initial screening. Three types of solid-phase extraction (SPE) cartridges that are HLB, MAX, and MCX (6cc, 500 mg) were used, enabling a three-tier selective mechanism that incorporates reverse-phase, anion exchange, and cation exchange to effectively remove PPCPs from wastewater. The HLB cartridge was used to remove mid-polar to polar PPCPs which are neutral in nature, MAX cartridge was used to bind anionic PPCPs, and MCX cartridge was used to bind cationic PPCPs. These cartridges were stacked sequentially in the order MAX (top), MCX (middle), and HLB (bottom) using cartridge-holding adapters. The stacked cartridges were conditioned with 10 mL of methanol and equilibrated with 10 mL of ASTM type I water. Then, 4 L of pooled wastewater sample was slowly passed through the cartridges, and cartridge-passed wastewater was directly collected at the bottom in polypropylene bottles and stored at 2–8 °C. After each 1-L wastewater sample was loaded, the cartridges were washed twice with 10 mL of methanol and then re-equilibrated with 20 mL of ASTM type I water.3.5. Evaluation of SPE Cartridges for Extraction Efficiency

### 3.5. Evaluation of SPE Cartridges for Extraction Efficiency

The solid-phase extraction efficiency was evaluated using three different 96-well plate SPE cartridges to compare their elution profiles and extraction efficiencies. The selected three types of SPE cartridges (MCX, HLB, and MAX) were preconditioned with 1 mL methanol. Further cartridges were equilibrated with 1 mL of ASTM type I water (HLB), 1 mL of 2% formic acid in water (MCX), and 1 mL of 5% ammonium hydroxide in water (MAX). Screened wastewater samples spiked at MQC level (*n* = 4) were passed through all three types of cartridges. All three types of cartridges were washed with the corresponding solvents: 1 mL of ASTM type I water (HLB), 1 mL of 2% formic acid in water (MCX), and 1 mL of 5% ammonium hydroxide in water (MAX). The cartridges were dried under vacuum for 10 min at 1000 mbar, and analytes were eluted with 500 µL of elution solvents: methanol–acetonitrile (70:30 *v*/*v*) for HLB, 2% formic acid in methanol–acetonitrile (70:30 *v*/*v*) for MAX, and 5% ammonium hydroxide in methanol–acetonitrile (70:30 *v*/*v*) for MCX. The eluents were reconstituted with 500 µL of ASTM type I water, vortex-mixed, and analyzed by LC-MS/MS.

### 3.6. Sample Extraction Automation

**Figure 6:**
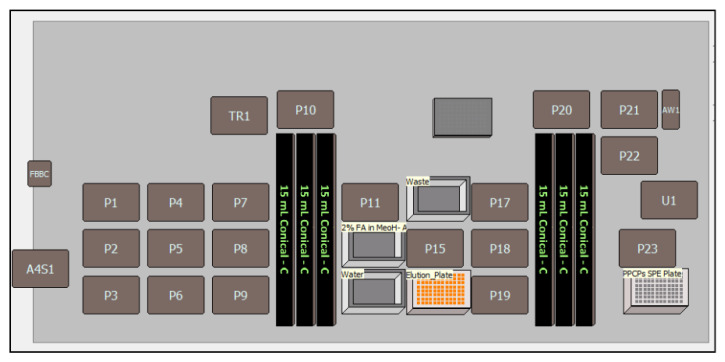
P1 to P19—Alps to keep SPE plate and reservoirs, AW1 is washing and rinsing station for 8-channel pipette arms, tube rack1 to 6 (15 mL conical) to load the samples, A4S1 is elution plate sealer, TR1 is diverting valve for SPE solvent selection, and P24 (1 SPE plate) station for SPE 96-well plate parking location for positive pressure SPE run.

Sample preparation was automated using Biomek i7 Workstation, equipped with automated 8-channel pipetting robotic arm, positive pressure SPE extractor and Multidrop Combi. The process of sample extraction begins with the user placing SPE plates, solvent reservoirs, and solvents (to conduct conditioning, equilibration, washing, and elution steps) on the workstation deck as guided by the software (Figure 6). The user then selects the tubes containing the samples and controls in the software to provide the pipetting and dispensing locations to the robotics. After the above tasks, the pre-programmed MAX SPE method was selected to run the complete extraction protocol. All the reagents were dispensed into full plates using the 8-channel pipetting tools and by Multidrop Combi dispenser by using diverting valve selection through software.

Pre-centrifuged 10 mL of wastewater samples were transferred to 15 mL polypropylene centrifuge tubes using a Hamilton robotics system. To these samples, 100 µL of a deuterated internal standard mix and 400 µL of an extraction buffer (5% ammonia in ASTM type I water) were added. Then samples, calibration controls (CCs), and quality controls (QCs) were mixed thoroughly using a Benchmark multi-tube vortexer. The prepared samples along with CC and QC samples were then loaded onto the Biomek i7 Workstation for SPE. Table 2 outlines the steps for the automated extraction of PPCPs in wastewater.

### 3.7. Estimation of Measurement Uncertainty (MU)

Measurement Uncertainty (MU) is associated with the result of a measurement, and it characterizes the dispersion of values that could be attributed to the measurand. Further, MU for the targeted analytes is calculated referring to the recently published research paper [49] following the approach of grouping the uncertainty components into two categories, that is, type A and type B. Type A uncertainty was associated with repeatability of the results produced using Biomek i7 automated protocol, while the type B uncertainty corresponded to factors such as standard purity, stock standard preparation, sample volume, volumetric flask, and pipetting volume. Calculated standard MU is converted to relative standard uncertainty (RSU), and thereafter, the square root of sum of squares of individual RSUs were combined to calculate the combined uncertainty. Combined uncertainty was then used to calculate expanded uncertainty using a coverage factor at a 95% confidence level [50,51].

### 3.8. Data Analysis, Calculation, and Representations

Data analysis was performed using TargetLynx module of MassLynx V4.2. and Microsoft Office 365 Excel worksheet was used for calculation of accuracy, repeatability, and intermediate repeatability as % Relative Standard Deviation (RSD) and Relative Percent Deviation (RPD), estimated LOD, LOQ, and plotting charts for data representation. The results of the extraction efficiency of three different SPE cartridges were statistically analyzed using SPSS 30.0 statistics software (IBM SPSS Statistics Version 30.0.0.0 (172), 2024, Armonk, NY, USA) for one-way Analysis of Variance (ANOVA). Online tools were used for evaluation of method greenness and its applicability.

### 3.9. Evaluation of the Method Greenness and Its Applicability

Given the growing emphasis on sustainable environments and green chemistry principles, various approaches have been developed to assess method greenness and practicality. The proposed method was evaluated for its greenness using two tools: Green Analytical Procedure Index (GAPI) and AGREE analytical greenness (AGREE), and its applicability for high-throughput analysis was assessed using Blue Applicability Grade Index (BAGI). GAPI is a semi-quantitative tool used to evaluate the environmental impact of analytical methods, considering stages such as sampling, sample preparation, reagents/chemicals, energy consumption, and other factors. It uses a color-coded pentagram (green, yellow, and red) to indicate environmental impact. Its latest version, ComplexMoGAPI was used to assess method greenness along with AGREE analytical greenness calculator [32,33]. AGREE evaluates the method on 12 principles of Green Analytical Chemistry, which are sampling procedure, sample volume, positioning of analytical device, number of sample preparation steps, sample preparation technique, derivatization, amount of waste generation, number of analytes in single run, instrument type, source of reagent, reagent toxicity, and operators’ safety. The Blue Applicability Grade Index (BAGI) is used to assess the practicality of analytical methods, assigning scores between 25 and 100, with higher scores indicating greater practicality [34].

## 4. Conclusions

The study concludes that the newly developed automated robotic system effectively overcomes the limitations of traditional WBE methods. The method demonstrated excellent specificity, sensitivity, precision, and accuracy. The maximum interfering peak was observed in the case of 1, 7 Dimethylxanthine (14.79%), which is well below the acceptable tolerance of 30% [47]. Furthermore, it was observed that six PPCPs were affected the most due to matrix effect, namely Minocycline (53.68%), followed by Erythromycin (38.29%), Cotinine (30.53%), Ranitidine (22.29%), Azithromycin (22.12%), and Ciprofloxacin (21.62%). This observation highlighted the necessity to use blank control wastewater matrix for plotting a procedural calibration curve to address matrix effects, as recommended in method validation guidelines [47]. Therefore, a novel approach was developed to prepare wastewater control matrix from routine wastewater samples using selective cartridges. The method also exhibited reliability in precision and accuracy trials conducted during validation, Blind Testing (BT), and Intra-Laboratory Comparison (ILC). The optimized automated MAX 96-well plate SPE protocol (automated extraction of 96 samples within 2 h and 40 min) and 12 min LC-MS/MS run time allows high-throughput analysis of up to 100 samples in 24 h. The optimized method also received high rankings in greenness evaluations (ComplexMoGAPI and AGREE) and an impressive BAGI score for practicality. Analysis with previously established conventional methods is typically restricted to smaller subsets of PPCPs, requiring multiple extraction steps along with chromatographic and mass spectrometric detection techniques. By integrating high-throughput sample preparation using a Biomek i7 Workstation with MAX 96-well plate SPE and LC-MS/MS detection, the method reliably and efficiently analyses 69 PPCPs in a single analytical protocol. The reported method, when integrated with AI-driven tools for real-time wastewater monitoring, provides an advanced framework for large-scale, year-round epidemiological surveillance. Spatial tagging of PPCPs concentrations and trend visualization streamlines routine sewage sample analysis across locations. This novel methodology improves detection efficiency for various PPCPs and contributes to public health and environmental management efforts by facilitating accurate, prompt, and scalable wastewater surveillance.

## Figures and Tables

**Table 1:** MRM transitions for targeted PPCPs and Internal Standards (IS) and Results for Extraction Efficiency (EE) study.

Sr. No.	Name of the PPCPs (Ionization)	Class of PPCPs	Details of MRM Parameters, Retention Time (RT), and Ion Ratios (PPCPs and IS)											Evaluation of SPE Efficiency of Targeted PPCPs (Average Area at MQC, *n* = 4)		
			Parent Ion	Product Ion (Q1, Q2)	CV (V)	CE (Q1, Q2) (eV)	Within Batch Stability (*n* = 26)		Details of the Internal Standard (IS) Used for Quantification							
							Ion Ratio (Q2/Q1 ± % RSD)	RT ± Stdev	Name of IS Used for Quantification	MRM Transition of IS	RT ± Stdev (IS)	CV (V)	CE (eV)	MAX	HLB	MCX
1	Ciprofloxacin	**Fluoroquinolones**	332.1	288.1, 314.1	35	22, 18	0.9802 ± 3.39	3.11 ± 0.007	Flumequine-13C3	265.1 > 247.03	5.84 ± 0.006	30	15	1,056,301 ^a^	645,745 ^b^	143,687 ^c^
2	Clinafloxacin		366.0	304.94, 347.79	15	20, 18	0.4452 ± 9.69	3.56 ± 0.012	Flumequine-13C3	265.1 > 247.03	5.84 ± 0.006	30	15	414,501 ^a^	300,808 ^b^	88,168 ^c^
3	Enrofloxacin		360.3	316.16, 245.11	12	19, 25	0.5257 ± 5.18	3.26 ± 0.013	Flumequine-13C3	265.1 > 247.03	5.84 ± 0.006	30	15	1,255,182 ^a^	930,841 ^b^	295,111 ^c^
4	Flumequine		262.1	201.96, 125.95	15	31, 45	0.3903 ± 3.05	5.84 ± 0.007	Flumequine-13C3	265.1 > 247.03	5.84 ± 0.006	30	15	2,210,158 ^a^	1,648,106 ^b^	665,288 ^c^
5	Lomefloxacin		352.1	265.02, 236.93	44	22, 34	0.3844 ± 4.40	3.22 ± 0.013	Flumequine-13C3	265.1 > 247.03	5.84 ± 0.006	30	15	1,114,359 ^a^	979,694 ^b^	280,779 ^c^
6	Norfloxacin		320.1	233.05, 204.83	15	24, 30	0.3726 ± 9.92	3.05 ± 0.005	Flumequine-13C3	265.1 > 247.03	5.84 ± 0.006	30	15	250,767 ^a^	132,066 ^b^	15,024 ^c^
7	Ofloxacin		362.1	261.02, 218.62	44	34, 26	0.0654 ± 8.84	3.04 ± 0.003	Flumequine-13C3	265.1 > 247.03	5.84 ± 0.006	30	15	1,400,463 ^a^	1,106,513 ^b^	310,326 ^c^
8	Oxolinic Acid		262.1	243.92, 159.96	25	20, 36	0.1559 ± 4.13	4.75 ± 0.005	Flumequine-13C3	265.1 > 247.03	5.84 ± 0.006	30	15	3,521,015 ^a^	2,622,939 ^b^	308,513 ^c^
9	Sarafloxacin		386.1	342.18, 299.09	35	18, 25	0.8187 ± 3.72	3.47 ± 0.014	Flumequine-13C3	265.1 > 247.03	5.84 ± 0.006	30	15	239,738 ^a^	146,017 ^b^	66,503 ^c^
10	Azithromycin	**Macrolides**	749.5	158.04, 82.98	45	40, 38	0.8659 ± 4.99	3.88 ± 0.004	Azithromycin-13C D3	753.7 > 115.92	3.88 ± 0.006	35	36	1,346,648 ^c^	2,792,538 ^b^	3,235,748 ^a^
11	Clarithromycin		748.3	590.14, 158.09	20	16, 16	1.1556 ± 6.67	5.83 ± 0.005	Roxithromycin-D7	844.7 > 157.99	5.91 ± 0.002	35	27	4,986,458 ^b^	6,314,206 ^a^	4,720,949 ^c^
12	Erythromycin (Negative)		734.4	157.98, 576.37	40.0	32, 18	0.4046 ± 7.55	5.18 ± 0.005	Erythromycin 13C D3	738.4 > 162.4	5.18 ± 0.005	15	30	697,088 ^b^	1,088,683 ^a^	450,116 ^c^
13	Erythromycin Anhydrate		716.4	158.0, 116.0	33.0	45, 30	0.5518 ± 2.85	5.58 ± 0.005	Erythromycin 13C D3	738.4 > 162.4	5.18 ± 0.005	15	30	7,158,761 ^c^	10,217,700 ^a^	8,098,076 ^b^
14	Lincomycin		407.1	125.99, 359.12	38.0	24, 18	0.0858 ± 4.97	2.89 ± 0.004	NA	NA	NA	NA	NA	3,781,706 ^a^	1,610,207 ^b^	3,905,783 ^a^
15	Roxithromycin		837.5	679.43, 158.04	44.0	20, 34	0.5391 ± 4.10	5.94 ± 0.006	Roxithromycin-D7	844.7 > 157.99	5.91 ± 0.002	35	27	4,139,132 ^c^	7,468,155 ^a^	6,084,984 ^b^
16	Virginiamycin		526.1	508.13, 355.05	30.0	12, 17	0.4833 ± 5.35	6.61 ± 0.003	Roxithromycin-D7	844.7 > 157.99	5.91 ± 0.002	35	27	2,320,324 ^b^	6,819,360 ^a^	7,299,741 ^a^
17	Tylosin		916.5	174.1, 101.1	45.0	40, 45	0.2002 ± 6.57	5.41 ± 0.005	NA	NA	NA	NA	NA	2,639,936 ^b^	5,072,795 ^a^	2,023,093 ^c^
18	Ampicillin	**Penicillins**	350.0	105.95, 159.94	28.0	16, 10	0.3503 ± 4.08	3.07 ± 0.006	NA	NA	NA	NA	NA	74,200 ^c^	281,386 ^a^	235,753 ^b^
19	Cloxacillin		436.2	160.0, 277.0	50.0	22, 40	NA	2.21 ± 0.006	NA	NA	NA	NA	NA	722,367 ^c^	1,060,749 ^b^	1,327,762 ^a^
20	Cefotaxime		456.0	395.89, 323.92	25.0	10, 14	0.6454 ± 3.45	3.77 ± 0.008	NA	NA	NA	NA	NA	586,648 ^c^	2,698,762 ^b^	4,045,124 ^a^
21	Oxacillin		402.3	143.97, 185.98	69.0	20, 15	0.3323 ± 5.57	6.49 ± 0.005	NA	NA	NA	NA	NA	2,009,376 ^a^	1,988,415 ^a^	2,339,547 ^b^
22	Penicillin G		335.0	217.13, 220.14	61.0	14, 13	0.2187 ± 3.59	5.65 ± 0.005	NA	NA	NA	NA	NA	3,299,258 ^b^	3,444,161 ^ab^	3,667,462 ^a^
23	Penicillin V		351.1	106.00, 160.01	30.0	20, 14	0.3252 ± 6.85	3.06 ± 0.006	NA	NA	NA	NA	NA	8752 ^c^	33,885 ^a^	28,001 ^b^
24	1 7 Dimethylxanthine	**Other PPCPs**	181.1	124.00, 68.92	30.0	18, 18	0.1207 ± 13.19	2.84 ± 0.006	NA	NA	NA	NA	NA	23,050,434 ^b^	27,725,049 ^a^	23,068,096 ^b^
25	Acetaminophen		152.1	110.14, 64.84	35.0	14, 25	0.1474 ± 4.13	2.70 ± 0.005	Acetaminophen-13C2	155.0 > 111	2.70 ± 0.005	15	16	53,102,347 ^c^	62,178,936 ^a^	56,092,384 ^b^
26	Albuterol		240.2	147.99, 165.94	24.0	16, 12	0.2856 ± 3.33	2.39 ± 0.003	NA	NA	NA	NA	NA	2,656,223 ^a^	324,846 ^c^	2,443,745 ^b^
27	Caffeine		195.0	137.97,123.2	15.0	20, 20	0.0164 ± 6.32	3.24 ± 0.009	Caffeine-13C3	198 > 140	3.24 ± 0.008	20	15	4,528,989 ^b^	5,095,626 ^a^	4,440,410 ^b^
28	Carbadox		263.0	230.8, 129.88	30.0	13, 20	0.8011 ± 5.64	3.53 ± 0.009	NA	NA	NA	NA	NA	889,938 ^b^	701,716 ^c^	1,156,898 ^a^
29	Carbamazepine		237.0	178.9, 165.04	38.0	34, 36	0.7634 ± 3.25	5.91 ± 0.006	NA	NA	NA	NA	NA	1,356,458 ^b^	1,310,734 ^b^	1,427,801 ^a^
30	Cimetidine		253.1	158.97, 94.96	26	12, 24	1.0375 ± 3.03	2.41 ± 0.005	Cimetidine-D3	256.1 > 162.04	2.41 ± 0.005	25	14	1,531,346 ^a^	380,811 ^b^	1,479,030 ^a^
31	Cotinine		176.7	79.86, 97.86	35	20, 20	0.2922 ± 11.53	2.30 ± 0.010	Cotinine-D3	180 > 101	2.30 ± 0.008	15	16	15,740,823 ^b^	1,712,875 ^c^	18,535,503 ^a^
32	Dehydro Nifedipine		344.9	283.98, 267.84	94	28, 28	0.3650 ± 4.32	6.47 ± 0.005	NA	NA	NA	NA	NA	1,033,433 ^a^	929,161 ^b^	1,088,647 ^a^
33	Digoxigenin		391.5	355.3, 373.3	30	15, 10	0.6746 ± 2.82	4.55 ± 0.005	NA	NA	NA	NA	NA	653,080 ^c^	769,604 ^b^	911,624 ^a^
34	Digoxin		803.3	282.9	49	45	NA	6.33 ± 0.003	NA	NA	NA	NA	NA	33,110 ^a^	25,353 ^c^	27,905 ^b^
35	Diltiazam		415.1	177.95, 149.92	36	24, 28	0.1044 ± 13.18	4.94 ± 0.006	Diltiazam-D3	418.2 > 177.9	4.93 ± 0.006	25	22	2,965,188 ^c^	5,532,001 ^b^	5,924,272 ^a^
36	Diphenhydramine		256.2	166.97, 151.88	15	12, 32	0.2653 ± 2.88	4.63 ± 0.006	Diphenhydramine-D3	259.2 > 166.97	4.63 ± 0.006	20	20	2,445,338 ^b^	2,827,255 ^a^	2,904,373 ^a^
37	Fluoxetine		310.2	251.99, 163.05	30	17, 17	0.9506 ± 3.39	6.83 ± 0.004	Fluoxetine-D5	315.2 > 43.88	5.57 ± 0.006	25	8	1,720,777 ^a^	1,303,781 ^b^	1,828,129 ^a^
38	Gemfibrozil (Negative)		249.0	121.0, 127.0	25	13, 10	0.0732 ± 1.50	8.22 ± 0.005	NA	NA	NA	NA	NA	902,455 ^a^	536,656 ^c^	730,348 ^b^
39	Ibuprofen (Negative)		205.0	161.02	20	8.0	NA	7.57 ± 0.007	NA	NA	NA	NA	NA	195,532 ^a^	173,539 ^b^	196,722 ^a^
40	Naproxen		231.1	184.99, 169.97	18	12, 24	0.3163 ± 13.33	6.69 ± 0.005	NA	NA	NA	NA	NA	1,468,498 ^a^	799,771 ^b^	1,508,501 ^a^
41	Norgestimate		370.2	123.97, 90.99	40	32, 48	0.6474 ± 4.69	8.27 ± 0.007	NA	NA	NA	NA	NA	1,030,506 ^a^	750,127 ^b^	1,213,558 ^a^
42	Ormetoprim		275.0	123.02, 259.08	30	25, 25	0.7967 ± 3.52	3.13 ± 0.006	NA	NA	NA	NA	NA	2,624,786 ^ab^	2807173 ^a^	2561420 ^b^
43	Ranitidine		315.1	175.99, 124	20	17, 17	0.2723 ± 4.92	2.45 ± 0.005	Ranitidine-D6	321.2 > 176.05	2.44 ± 0.005	30	16	2,757,507 ^b^	857,502 ^c^	3,015,978 ^a^
44	Thiabendazole		202.0	130.96, 174.95	31	40, 40	0.2368 ± 6.10	3.42 ± 0.011	NA	NA	NA	NA	NA	1,197,504 ^a^	1,190,961 ^a^	1,311,527 ^a^
45	Warfarin (Negative)		307.1	161.0, 250.0	35	20, 25	0.5237 ± 1.11	6.84 ± 0.004	Warfarin-D5	312.1 > 160.82	6.82 ± 0.00	15	20	6431222 ^a^	5217781 ^b^	6727270 ^a^
46	Sulfamethizole	**Sulfonamides**	271.1	156.0, 92.0	30	15, 25	0.6451 ± 3.36	3.61 ± 0.010	Sulfamethizole-13C6	276.90 >162.02	3.61 ± 0.001	25	14	2,010,358 ^c^	2,261,471 ^b^	2,490,050 ^a^
47	Sulfachloropyridazine		285.1	155.93, 91.94	30	13, 29	0.6132 ± 4.50	4.01 ± 0.007	NA	NA	NA	NA	NA	1,943,737 ^a^	2,071,357 ^a^	1,986,850 ^a^
48	Sulfadiazine		251.0	156.0, 92.0	30	15, 27	0.8029 ± 4.48	2.93 ± 0.006	Sulfadiazine-13C6	257.0 > 162.04	2.93 ± 0.005	20	15	1,551,625 ^b^	2,002,372 ^a^	1,627,019 ^b^
49	Sulfadimethoxine		311.1	156.0, 92.0	36	20, 32	0.4828 ± 3.19	4.87 ± 0.004	Sulfadimethoxine-D6	317 > 162.15	4.84 ± 0.005	25	22	3,313,191 ^a^	3,058,455 ^a^	3,043,851 ^a^
50	Sulfamerazine		265.1	156.0, 92.0	35	15, 25	0.9283 ± 11.57	3.33 ± 0.008	Sulfamearazine_13C6	271. 1> 171.9	3.33 ± 0.008	25	16	1,334,396 ^b^	1,581,985 ^a^	1,409,520 ^b^
51	Sulfamethazine		279.1	186.0, 124.1	40	15, 25	0.5960 ± 8.50	3.70 ± 0.007	Sulfamethazine-13C6	285 > 186.03	3.69 ± 0.007	25	16	2,326,698 ^b^	2,511,412 ^a^	2,372,135 ^ab^
52	Sulfamethoxazole		254.1	156.0, 92.0	30	15, 25	0.9546 ± 3.97	4.14 ± 0.007	Sulfamethoxazole-13C6	260.1 > 161.96	4.14 ± 0.007	35	16	1,447,175 ^b^	1,601,116 ^a^	1,536,744 ^ab^
53	Sulfathiazole		256.0	156.0, 92.0	31	15, 25	0.6875 ± 5.78	3.08 ± 0.003	Sulfamethoxazole-13C6	260.1 > 161.96	4.14 ± 0.007	35	16	2,110,959 ^b^	2,716,660 ^a^	2,338,768 ^b^
54	Trimethoprim		291.1	229.99, 123	45	22, 24	0.8770 ± 3.38	2.96 ± 0.006	Trimethoprim 13C3	294 > 233	2.97 ± 0.006	45	25	1,501,819 ^a^	1,594,025 ^a^	1,525,977 ^a^
55	4 Epioxytetracycline	**Tetracyclines**	460.9	425.9, 443.15	28	18, 13	0.2978 ± 3.92	3.20 ± 0.009	NA	NA	NA	NA	NA	8,026,750 ^b^	8,922,119 ^a^	53,390 ^c^
56	4 Epianhydrochlortetracycline		461.0	443.91, 97.88	25	20, 35	0.2380 ± 10.52	5.22 ± 0.006	NA	NA	NA	NA	NA	1,962,967 ^a^	2,263,661 ^a^	375,931 ^b^
57	4 Epianhydrotetracycline		427.2	410.2, 154.0	36	18, 34	0.0367 ± 5.39	4.45 ± 0.006	NA	NA	NA	NA	NA	17,573,393 ^a^	19,431,498 ^a^	1,535,024 ^b^
58	4 Epichlortetracycline		479.3	461.85, 97.88	25	18, 41	0.0543 ± 4.89	3.35 ± 0.012	NA	NA	NA	NA	NA	11,270,071 ^a^	9,947,026 ^b^	2,860,411 ^c^
59	4 Epitetracycline		445.5	410.0, 154.0	25	20, 25	0.0741 ± 5.99	3.12 ± 0.004	NA	NA	NA	NA	NA	12,960,601 ^b^	17,010,339 ^a^	107,167 ^c^
60	Anhydro Chlortetracycline		461.0	443.92, 153.9	12	25, 25	0.7813 ± 11.68	6.68 ± 0.005	NA	NA	NA	NA	NA	1,552,990 ^a^	399,176 ^b^	374,544 ^b^
61	Anhydrotetracycline		427.2	410.2, 154.0	36	16, 34	0.2444 ± 2.21	5.52 ± 0.005	NA	NA	NA	NA	NA	31,317,995 ^a^	11,519,591 ^b^	3,591,401 ^c^
62	Chlortetracycline		479.3	461.85, 97.88	25	18, 41	1.2606 ± 7.42	4.25 ± 0.004	NA	NA	NA	NA	NA	1,659,364 ^b^	3,486,243 ^a^	17,343 ^c^
63	Demeclocycline		465.1	430.01, 153.88	2	20, 28	0.9855 ± 4.01	3.82 ± 0.007	NA	NA	NA	NA	NA	3,952,343 ^b^	6,117,631 ^a^	35,931 ^c^
64	Doxycycline		445.1	154.0, 428.1	20	28, 15	0.1852 ± 4.38	3.51 ± 0.008	NA	NA	NA	NA	NA	11,572,856 ^b^	16,328,640 ^a^	101,422 ^c^
65	Isochlortetracycline		479.3	461.85, 97.88	25	18, 41	0.1985 ± 7.75	3.69 ± 0.009	NA	NA	NA	NA	NA	19,874,394 ^a^	15,761,242 ^b^	7,370,522 ^c^
66	Miconazole		417.0	159.0, 161.0	20	19, 19	0.9912 ± 3.13	6.69 ± 0.001	NA	NA	NA	NA	NA	1,911,547 ^c^	3,001,115 ^b^	4,197,732 ^a^
67	Minocycline		458.0	441.0, 352.0	20	15, 30	0.2533 ± 6.56	3.62 ± 0.010	NA	NA	NA	NA	NA	3,065,017 ^b^	4,035,484 ^a^	67,515 ^c^
68	Oxytetracycline		461.0	426.03, 336.99	30	15, 28	0.2216 ± 2.93	3.44 ± 0.012	NA	NA	NA	NA	NA	11,368,429 ^b^	12,319,767 ^a^	141,176 ^c^
69	Tetracycline		445.5	410.0, 154.0	25	20, 25	0.8208 ± 2.86	3.51 ± 0.010	NA	NA	NA	NA	NA	14,294,950 ^b^	20,725,639 ^a^	127,031 ^c^

NA—Not Applicable. Q1—Quantifier Ion, Q2—Qualifier Ion, CV—Cone Voltage, CE—Collision Energy, MRM—Multiple Residue Monitoring, RT—Retention Time, RSD—Relative Standard Deviation, IS—Internal Standard, RPD—Relative Percentage Deviation, MQC—Mid Quality Control, MCX—Mixed-Mode Cation Exchange, HLB—Hydrophilic–Lipophilic Balance, MAX—Mixed-Mode Anion Exchange; mean in each row with different superscripts for evaluation of extraction efficiency (a > b > c) are significantly different (*p* < 0.05) from each other.

**Table 2:** Analytical conditions for automated extraction and detection of PPCPs in wastewater.

Automated extraction of PPCPs in wastewater using Biomek i7 Workstation	**Steps**	**Sample Preparation Task**		
	Sample Preparation	Unknown wastewater samples and spiked (QC) wastewater samples with ISTD (0.100 mL) and extraction buffer (0.400 mL of 5% ammonia in ASTM type I water for PPCPs) were loaded in the 15 mL conical tube racks (samples 10.0 mL) and precleared by centrifugation at 4500 rpm in a model (Eppendorf).		
	Conditioning	Condition the MAX 96-well plate cartridge with 1.0 mL 100% MeOH.		
	Equilibration	Equilibrate the cartridge with 1.0 mL of 100% ASTM type I water.		
	Sample Loading	Load 1.5 mL of the sample and quality controls three times (4.5 mL) on respective MAX 96-well plate cartridge.		
	Washing	Wash 96 wells with 1.0 mL of 5% ammonia in ASTM type I water. Dry the cartridge at 1000 mbar for up to 10 min.		
	Elution	Elute 96-well plate with 0.5 mL of 2% formic acid in methanol–acetonitrile (50:50, *v*/*v*).		
	Dilution, Well Plates Sealing	Dilute the final elute sample with 0.750 mL of water and seal the cartridge plate with a4S 4titude automated role heat sealer and vortex mixture at lower rpm.		
	LC-MS/MS	Load sealed 96-well plate cartridge LC-MS/MS autosampler and inject 4 μL of sample volume.		
Equipment Details	Ion Source	Z Spray XEVO Ion Source		
	Pump	Acquity UPLC-I Class Plus		
	Autosampler	FTN Sample Manager		
	Column Oven	Acquity UPLC Column Heater		
	LC Column	ACQUITY Premier BEH C18 Column, 1.7 µm, 2.1 mm × 100 mm		
LC Parameters	Mobile Phase A	0.1% acetic acid in water		
	Mobile Phase B	0.1% acetic acid in methanol–acetonitrile [50/50, *v*/*v*]		
	Sample Purge	Methanol–acetonitrile–water–IPA [1:1:1:1, *v*/*v*/*v*/*v*] with 0.5% acetic acid		
	Sample Wash	Methanol–acetonitrile–water–IPA [1:1:1:1, *v*/*v*/*v*/*v*] with 0.5% acetic acid		
	Seal Wash	Methanol–water [10/90, *v*/*v*]		
	Flow Rate	0.350 mL/minute		
	Column Oven	55 ± 5 °C		
	Sample Manager	10 ± 3 °C		
	Injection	4.0 µL volume		
LC Gradient	Flow	Time (min)	Pump A %	Pump B %
	0.35	Initial	100.00	0.00
	0.35	0.60	100.00	0.00
	0.35	2.00	80.00	20.00
	0.35	5.00	50.00	50.00
	0.35	5.50	50.00	50.00
	0.35	5.60	30.00	70.00
	0.35	8.00	10.00	90.00
	0.35	9.00	10.00	90.00
	0.35	9.10	2.00	98.00
	0.35	10.00	2.00	98.00
	0.35	10.10	100.00	0.00
	0.35	12.00	100.00	0.00
MS Parameters	Mode and polarity		ESI +/−	
	Scan type		(Segmented MRM)	
	Source temperature (°C)		150.00	
	Disolvation gas temp. (°C)		600.00	
	Disolvation gas flow (L/h)		1100.00	
	Cone gas flow (L/h)		150.00	
	Capillary voltage (kV)		1.20 (Positive)/2.80 (Negative)	
	Nebulizer gas flow (bar)		7.00	

**Table 3:** Results for method validation parameters for PPCPs.

Sr. No.	Name of the PPCPs	Class (PPCPs)	Results for Method Validation															
			Specificity (%)	Matrix Effect (%)	Linearity Range ( µg/L)	Coefficient of Determination (r^2^) and % Deviation from Back-Calculated Concentration of Linear Calibration Curve (Average of Results from Day 1, 2, and 3 Validation Trials)									E-LOD ( µg/L)	E-LOQ ( µg/L)	T-LOQ ( µg/L)	MU (@ Mean Calculated Concentration at LOQ ± MU) µg/L
						r^2^	L1	L2	L3	L4	L5	L6	L7	L8				
1	Ciprofloxacin	Fluoroquinolones	0.85	21.62	0.50–20.0	0.9870	5.73	−16.03	5.13	8.68	8.46	−0.71	−4.76	−6.56	0.022	0.067	0.500	0.530 ± 0.025
2	Clinafloxacin		0.99	8.61	0.10–4.0	0.9937	−0.67	−0.33	1.00	5.67	1.20	−4.84	−1.41	−0.88	0.011	0.032	0.100	0.114 ± 0.009
3	Enrofloxacin		0.34	15.36	0.05–2.0	0.9984	0.67	−2.33	−1.50	1.80	4.80	0.24	2.67	−7.07	0.006	0.018	0.050	0.053 ± 0.005
4	Flumequine		0.34	−1.86	0.05–2.0	0.9973	−2.67	5.33	−4.33	2.73	4.50	−1.92	−0.53	−3.75	0.009	0.027	0.050	0.050 ± 0.007
5	Lomefloxacin		1.49	2.82	0.05–2.0	0.9969	1.33	−3.00	−2.83	4.80	−0.97	−0.80	0.51	0.27	0.009	0.028	0.050	0.054 ± 0.008
6	Norfloxacin		6.32	18.17	0.50–20.0	0.9835	7.07	−18.65	8.42	8.43	6.62	−1.50	−2.34	−6.57	0.044	0.135	0.500	0.554 ± 0.038
7	Ofloxacin		2.22	10.44	0.05–2.0	0.9923	4.00	−9.67	0.17	6.40	3.37	−2.80	0.38	−2.10	0.008	0.024	0.050	0.054 ± 0.005
8	Oxolinic Acid		0.79	0.68	0.05–2.0	0.9978	−4.00	12.33	−7.67	−0.13	1.20	−0.24	0.53	−1.85	0.004	0.012	0.050	0.051 ± 0.003
9	Sarafloxacin		1.48	5.84	0.10–4.0	0.9930	0.00	1.83	−5.67	7.97	3.68	−2.25	−1.74	−3.53	0.016	0.050	0.100	0.104 ± 0.013
10	Azithromycin	Macrolides	0.22	22.12	1.0–40.0	0.9838	−5.67	8.75	4.87	3.53	4.77	−3.60	−6.97	−5.68	0.280	0.848	1.000	0.997 ± 0.212
11	Clarithromycin		0.11	4.83	1.0–40.0	0.9933	3.90	−9.05	−2.83	−13.57	−6.50	13.63	1.60	10.12	0.240	0.727	1.000	1.092 ± 0.184
12	Erythromycin		0.00	38.29	1.0–40.0	0.9806	5.43	−5.28	−10.92	−4.67	−1.00	−0.82	7.00	10.26	0.171	0.517	1.000	1.019 ± 0.135
13	Erythromycin Anhydrate		0.02	−0.02	1.0–40.0	0.9742	−1.07	4.03	−3.83	−1.32	1.43	0.11	3.97	−3.35	0.241	0.731	1.000	1.131 ± 0.183
14	Lincomycin		0.12	1.09	0.05–2.0	0.9923	−3.33	3.50	−9.00	−0.73	2.37	−4.83	0.44	3.28	0.006	0.019	0.050	0.051 ± 0.005
15	Roxithromycin		0.00	2.20	1.0–40.0	0.9943	−2.20	−3.10	−7.58	−3.67	7.03	−2.54	−3.68	4.18	0.118	0.359	1.000	1.042 ± 0.095
16	Virginiamycin		1.19	1.78	1.0–40.0	0.9858	1.97	1.75	−2.91	−8.71	4.94	15.16	0.46	0.20	0.306	0.926	1.000	1.056 ± 0.242
17	Tylosin		0.00	3.78	1.0–40.0	0.9812	−0.97	7.32	−9.08	−15.58	1.54	−1.61	0.41	6.31	0.264	0.800	1.000	1.041 ± 0.199
18	Ampicillin	Penicillin	0.04	−3.33	0.50–20.0	0.9935	−4.33	7.13	2.33	6.76	−3.69	−1.43	−1.12	−5.58	0.107	0.325	0.500	0.530 ± 0.083
19	Cloxacillin		1.92	1.59	2.5–100.0	0.9724	−7.72	14.79	−0.55	10.42	−0.65	−6.84	−4.62	−4.82	0.703	2.131	2.500	2.616 ± 0.532
20	Cefotaxime		0.41	−0.39	1.0–40.0	0.9957	−1.77	4.77	−3.20	4.12	−6.170	0.47	5.71	−3.90	0.122	0.370	1.000	0.984 ± 0.097
21	Oxacillin		0.32	−6.39	0.50–20.0	0.9951	−0.07	6.67	−12.87	−4.24	4.563	−2.44	4.30	4.08	0.080	0.243	0.500	0.496 ± 0.061
22	Penicillin G		0.07	−2.34	0.50–20.0	0.9942	−4.67	−0.38	−5.27	−3.47	3.926	−3.17	1.50	1.66	0.058	0.175	0.500	0.484 ± 0.045
23	Penicillin V		1.35	−3.26	2.5–100.0	0.9891	−2.85	5.01	−0.84	9.65	−3.093	−3.56	−0.44	−3.88	0.647	1.959	2.500	2.874 ± 0.49
24	1 7 Dimethylxanthine	Others	14.79	−2.14	0.05–2.0	0.9980	−1.33	7.33	−7.50	−0.87	1.400	−0.35	2.24	−0.50	0.008	0.023	0.050	0.051 ± 0.005
25	Acetaminophen		9.47	−1.53	0.05–2.0	0.9960	−2.67	9.00	−6.33	−1.47	4.533	−1.09	−2.47	0.70	0.009	0.027	0.050	0.050 ± 0.008
26	Albuterol		0.08	0.40	0.05–2.0	0.9985	−4.00	9.33	−5.00	0.93	2.667	−0.99	−1.93	−1.57	0.005	0.015	0.050	0.054 ± 0.005
27	Caffeine		10.52	−1.40	0.5–20.0	0.9959	2.47	−5.15	−4.75	2.42	2.757	0.19	1.62	−1.31	0.066	0.200	0.500	0.505 ± 0.052
28	Carbadox		1.96	−2.50	0.10–4.0	0.9894	−8.67	10.50	−4.67	3.60	−3.500	−1.63	−0.91	−3.50	0.025	0.077	0.100	0.103 ± 0.02
29	Carbamazepine		0.35	−3.49	0.10–4.0	0.9977	−6.67	2.50	−2.17	7.23	−0.050	−1.43	−3.43	−7.36	0.016	0.048	0.100	0.103 ± 0.013
30	Cimetidine		3.46	−0.91	0.05–2.0	0.9953	−4.00	3.00	−8.50	−1.20	2.400	−0.16	−3.44	2.28	0.006	0.018	0.050	0.050 ± 0.005
31	Cotinine		5.47	30.53	0.10–4.0	0.9827	−0.67	6.33	−10.33	−1.30	8.117	−0.80	−4.02	2.73	0.021	0.064	0.100	0.104 ± 0.015
32	Dehydro Nifedipine		0.24	−2.36	0.10–4.0	0.9974	−2.00	8.33	−9.25	1.60	−0.967	0.59	1.37	0.32	0.013	0.041	0.100	0.101 ± 0.01
33	Digoxigenin		0.97	−6.19	0.10–4.0	0.9988	−5.33	1.25	−3.58	3.27	−1.967	−1.56	−2.46	−0.61	0.017	0.052	0.100	0.100 ± 0.013
34	Digoxin		1.59	2.76	2.5–100.0	0.9831	−7.17	14.12	13.10	8.18	−0.879	−0.71	−11.58	−15.52	0.444	1.345	2.500	2.565 ± 0.339
35	Diltiazam		1.90	−0.85	0.10–4.0	0.9946	−3.00	10.67	−7.42	−5.13	2.000	2.45	1.91	−1.31	0.029	0.087	0.100	0.102 ± 0.022
36	Diphenhydramine		0.39	0.93	0.05–2.0	0.9945	−2.00	−3.00	−9.50	−4.60	3.367	0.83	0.22	2.60	0.005	0.014	0.050	0.048 ± 0.003
37	Fluoxetine		0.25	−4.08	0.10–4.0	0.9935	−2.00	6.00	−5.42	5.27	4.033	1.67	−3.14	−6.23	0.013	0.039	0.100	0.093 ± 0.008
38	Gemfibrozil		0.08	−0.02	0.50–20.0	0.9994	−3.13	0.85	−2.78	−1.85	1.755	−0.92	1.57	1.02	0.049	0.148	0.500	0.508 ± 0.04
39	Ibuprofen		2.93	0.18	1.0–40.0	0.9977	−2.90	−2.47	−5.86	−1.15	0.640	0.22	−0.39	0.55	0.123	0.373	1.000	1.071 ± 0.098
40	Naproxen		0.93	−12.72	1.0–40.0	0.9881	9.60	10.58	−8.63	−1.77	−4.845	2.42	−8.57	8.23	0.189	0.573	1.000	1.062 ± 0.145
41	Norgestimate		0.71	−0.31	0.50–20.0	0.9631	2.73	7.37	−21.70	−16.23	−5.107	−4.57	13.69	16.09	0.093	0.283	0.500	0.473 ± 0.071
42	Ormetoprim		0.12	−4.90	0.05–2.0	0.9940	−3.33	1.50	−8.83	3.00	4.967	−3.23	−1.13	−2.15	0.006	0.018	0.050	0.055 ± 0.005
43	Ranitidine		0.25	22.29	0.10–4.0	0.9959	−1.33	5.33	−5.08	0.47	0.367	0.20	−1.32	1.55	0.016	0.047	0.100	0.108 ± 0.013
44	Thiabendazole		0.91	−2.65	0.10–4.0	0.9938	3.00	−8.25	−2.25	3.07	−0.100	0.53	1.14	0.26	0.033	0.099	0.100	0.111 ± 0.024
45	Warfarin		0.02	−0.24	0.50–20.0	0.9948	−6.73	−0.48	0.69	1.76	2.873	−2.05	−5.17	−5.27	0.018	0.054	0.500	0.524 ± 0.021
46	Sulfamethizole	Sulfonamides	2.89	−3.55	0.05–2.0	0.9907	13.33	4.33	−9.33	−0.87	2.800	0.56	0.93	0.12	0.006	0.020	0.050	0.05 ± 0.005
47	Sulfachloropyridazine		0.46	−3.44	0.05–2.0	0.9980	−2.00	8.67	−7.00	−0.47	−0.167	−1.55	0.64	2.53	0.006	0.018	0.050	0.048 ± 0.005
48	Sulfadiazine		0.42	−1.88	0.05–2.0	0.9971	−4.00	10.67	−5.50	0.13	0.567	−1.36	−2.96	2.23	0.009	0.027	0.050	0.050 ± 0.008
49	Sulfadimethoxine		0.33	−1.42	0.05–2.0	0.9981	−3.33	0.00	−7.67	−3.20	3.833	0.83	−1.93	0.90	0.008	0.023	0.050	0.051 ± 0.005
50	Sulfamerazine		0.66	−2.22	0.05–2.0	0.9889	−0.67	6.33	−8.83	1.60	3.533	7.44	−0.02	−8.52	0.012	0.038	0.050	0.051 ± 0.01
51	Sulfamethazine		1.41	−4.22	0.05–2.0	0.9951	−2.00	7.67	−7.83	−5.67	−3.133	−0.11	−1.22	11.52	0.008	0.025	0.050	0.049 ± 0.007
52	Sulfamethoxazole		0.87	−1.77	0.05–2.0	0.9961	−2.00	7.33	−7.00	−2.20	−0.300	3.79	0.44	−0.17	0.006	0.018	0.050	0.050 ± 0.005
53	Sulfathiazole		0.64	−2.06	0.05–2.0	0.9974	−4.67	14.67	−12.17	−0.47	−0.200	−0.67	6.04	−2.92	0.011	0.033	0.050	0.049 ± 0.008
54	Trimethoprim		0.78	−7.22	0.05–2.0	0.9981	−2.67	9.00	−8.67	−1.53	2.267	1.60	−2.13	1.62	0.003	0.010	0.050	0.049 ± 0.003
55	4 Epioxytetracycline	Tetracyclines	1.35	8.47	1.0–40.0	0.9882	1.10	1.07	−5.81	−2.24	−6.415	1.25	5.93	5.13	0.140	0.424	1.000	1.196 ± 0.134
56	4 Epianhydro Chlortetracycline		8.11	−4.23	1.0–40.0	0.9763	8.93	−12.33	−18.36	−5.13	−7.580	5.36	15.54	11.15	0.075	0.226	1.000	1.256 ± 0.070
57	4 Epianhydrotetracycline		3.26	−0.08	1.0–40.0	0.9750	0.53	6.65	−12.73	−10.02	−3.462	−1.93	8.04	12.94	0.123	0.374	1.000	1.221 ± 0.099
58	4 Epichlortetracycline		0.36	8.66	1.0–40.0	0.9925	−2.73	11.37	−11.65	−1.93	0.610	−0.38	2.81	1.91	0.174	0.528	1.000	1.096 ± 0.152
59	4 Epitetracycline		1.72	−0.33	1.0–40.0	0.9952	−0.13	2.13	−3.38	−1.71	−3.357	1.85	5.31	−0.73	0.198	0.599	1.000	1.181 ± 0.152
60	Anhydro Chlortetracycline		13.74	−3.05	1.0–40.0	0.9662	6.50	−8.90	−11.27	−8.49	−5.318	8.15	15.54	5.70	0.230	0.697	1.000	1.220 ± 0.179
61	Anhydrotetracycline		3.88	−4.85	1.0–40.0	0.9805	−0.30	5.68	−9.51	−2.03	−5.295	−3.61	7.18	7.86	0.085	0.257	1.000	1.233 ± 0.077
62	Chlortetracycline		3.90	−7.00	1.0–40.0	0.9779	2.93	−0.45	−10.58	−1.94	−7.475	0.67	6.86	10.00	0.218	0.662	1.000	1.208 ± 0.172
63	Demeclocycline		5.15	−6.97	1.0–40.0	0.9654	4.97	−5.18	−10.10	−0.38	−8.512	3.63	9.30	6.27	0.191	0.577	1.000	1.212 ± 0.148
64	Doxycycline		1.99	−1.23	1.0–40.0	0.9825	−1.17	5.47	−6.17	0.20	−3.197	−3.51	5.49	2.89	0.187	0.566	1.000	1.168 ± 0.147
65	Isochlortetracycline		0.74	−1.69	1.0–40.0	0.9727	1.67	2.72	−13.06	1.70	−6.35	1.92	4.65	6.77	0.200	0.607	1.000	1.120 ± 0.154
66	Miconazole		0.60	−1.94	0.50–20.0	0.9527	3.67	11.30	−18.48	−8.31	−1.61	−7.02	9.30	12.00	0.073	0.222	0.500	0.540 ± 0.057
67	Minocycline		5.09	53.68	1.0–40.0	0.9674	5.77	−4.87	−13.81	−3.67	2.56	−3.35	9.00	8.38	0.157	0.474	1.000	1.249 ± 0.122
68	Oxytetracycline		0.98	−0.71	1.0–40.0	0.9882	−0.50	5.35	−9.00	0.71	−5.02	0.23	4.73	3.50	0.089	0.270	1.000	1.172 ± 0.078
69	Tetracycline		1.99	−1.53	1.0–40.0	0.9820	−0.17	4.28	−8.19	0.75	−3.33	−0.51	2.23	4.95	0.211	0.640	1.000	1.179 ± 0.164

ME: Matrix Effect, E-LOD: Estimated Limit of Detection, E-LOQ: Estimated Limit of Quantification, T-LOQ: Targeted Limit of Quantification, MU: Measurement Uncertainty.

## Data Availability

Data sharing is not applicable as it is contained within the article.
